# Methyladenosine Modification in RNAs: Classification and Roles in Gastrointestinal Cancers

**DOI:** 10.3389/fonc.2020.586789

**Published:** 2021-02-01

**Authors:** Qinghai Li, Weiling He, Guohui Wan

**Affiliations:** ^1^Department of Gastrointestinal Surgery, the First Affiliated Hospital of Sun Yat-Sen University, Guangzhou, China; ^2^Center for Precision Medicine, Sun Yat-Sen University, Guangzhou, China; ^3^School of Pharmaceutical Sciences, Sun Yat-Sen University, Guangzhou, China

**Keywords:** RNA, methylation modification, m^6^A, m^6^Am, m^1^A, gastrointestinal cancers

## Abstract

Cellular ribonucleic acids (RNAs), including messenger RNAs (mRNAs) and non-coding RNAs (ncRNAs), harbor more than 150 forms of chemical modifications, among which methylation modifications are dynamically regulated and play significant roles in RNA metabolism. Recently, dysregulation of RNA methylation modifications is found to be linked to various physiological bioprocesses and many human diseases. Gastric cancer (GC) and colorectal cancer (CRC) are two main gastrointestinal-related cancers (GIC) and the most leading causes of cancer-related death worldwide. In-depth understanding of molecular mechanisms on GIC can provide important insights in developing novel treatment strategies for GICs. In this review, we focus on the multitude of epigenetic changes of RNA methlyadenosine modifications in gene expression, and their roles in GIC tumorigenesis, progression, and drug resistance, and aim to provide the potential therapeutic regimens for GICs.

## Introduction

With the deepening of genetics research and the emergence of epigenetics, many reversible chemical modifications have been identified. In RNAs, human cells undergo various forms of modification with different levels (1–3). The constitutive non-coding RNAs (ncRNAs) are known to contain larger number of pseudouridine (Ψ) and 2’-O-methylations (2’-OMe or Nm) modifications (1). In addition, various modifications are identified in the regulatory ncRNAs including small ncRNAs (sncRNAs), long ncRNAs (lncRNAs), and circular RNAs (circRNAs) and play important roles in metabolism and functions (4–7). However, owing to the spatiotemporal specificity of regulatory ncRNAs in various tissues, the detailed and conserved biological characteristics of most RNA modifications are unclear. As for mRNAs, internal methylation modifications have been recently revealed with help of the advanced detection and analysis technologies as well as the common modification of N^7^-methylguanosine (m^7^G) cap in the 5’ terminal region of mRNA (8–12). The most prevalent and crucial internal methylation form in mRNAs is N^6^-methyladenosine (m^6^A) modification that firstly identified in 1974 in eukaryotic cells (12–16), while the other major forms include N^6^,2’-O-dimethyladenosine (m^6^Am), N^1^-methyladenosine (m^1^A), 2’-OMe, and 5-methylcytosine (m^5^C).

Gastric cancer (GC) and colorectal cancer (CRC) are the most common gastrointestinal-related cancers (GICs). CRC is the fourth most commonly diagnosed cancer (6.1%) and the second leading cause of cancer death (9.2%) worldwide, while GC is the sixth diagnosed caner and the third cause of cancer death (8.2%) (17). In-depth research on molecular mechanisms in GICs can provide important insights in developing novel treatment strategies for GICs.

Recently, RNA methylation has been found to play critical roles in various bioprocesses including embryonic development, RNAs metabolism, gene expression regulation, and its aberrant regulation has been linked to many human diseases including cancer (18). Herein, we mainly focus on the multitude of epigenetic changes of RNA methlyadenosine modifications in gene expression, and their roles in GIC tumorigenesis, progression, and drug resistance, and aim to provide the potential therapeutic regimens for GICs.

## m^6^A Modification

### Biological Characteristics of m^6^A Modification

Although m^6^A is an “old” modification form that was firstly discovered in 1974 (13, 14), it had not gained enough attention until two breakthrough methods developed in 2011. The first breakthrough is the discovery of FTO (fat mass and obesity-associated protein), the first mammalian m^6^A demethylase in 2011 (19) and AlkB homolog 5 (ALKBH5), another demethylase in mouse fertility and spermatogenesis in 2013 (20), which proves and highlights that the m^6^A modification is a dynamic process and regulated by both methyltransferase and demethylase. The second breakthrough is that the transcriptome-wide distribution of m^6^A modification has been well revealed at ~100–200-nucleotide resolution in 2012 owing to the development of methylated RNA immunoprecipitation sequencing (MeRIP-seq or m^6^A-seq) technology (21, 22). Since then, other detection methods such as single-nucleotide resolution, antibody-independent, or isoform characterization analysis, have emerged as powerful tools for the m^6^A analysis. These tools mainly include site-specific cleavage and radioactive labeling followed by ligation-assisted extraction and thin layer chromatography (SCARLET) (23), m^6^A individual-nucleotide-resolution crosslinking and immunoprecipitation (miCLIP) (4), m^6^A level and isoform characterization sequencing (m^6^A-LAIC-seq) (24), deamination adjacent to RNA modification targets sequencing (DART-seq) (25), MAZTER-seq (26), m^6^A-sensitive RNA-endoribonuclease-facilitated sequencing (m^6^A-REF-seq) (27), m^6^A-lable-seq (28), m^6^A-SEAL (29), and the third-generation sequencing technologies (30). However, these methods have shortcomings such as inconvenient procedures (radioisotope p^32^), high cost, unavailability to distinguish m^6^A and m^6^Am, and detection limits of a certain motif.

**Figure 1:**
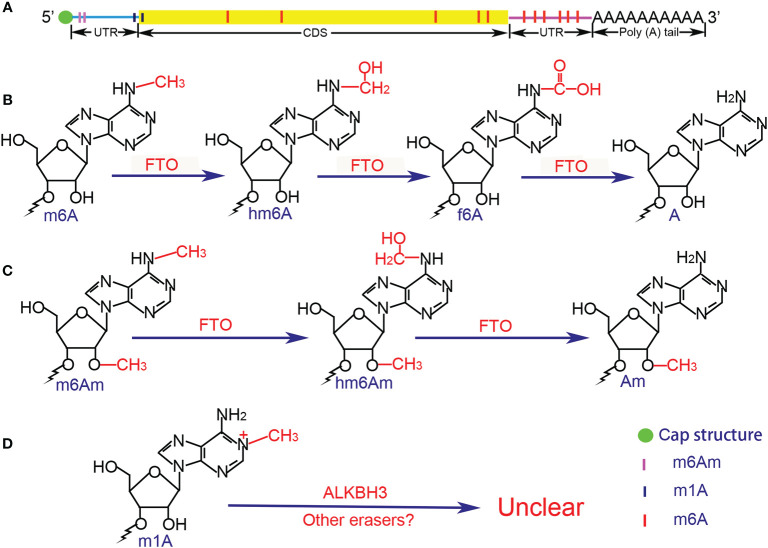
Distribution and chemical structure of methylation modifications. **(A)** m^6^Am and m^1^A are mainly enriched in the 5’UTR, whereas m^6^A is concentrated in the 3’UTR. **(B)** Demethylation of m^6^A is in a stepwise manner, the intermediate of hm^6^A is the direct oxidation product of m^6^A, while f^6^A is the further oxidized product of hm^6^A, and the final product is A. **(C)** The demethylation process of m^6^Am is similar to that of m^6^A, but the potential intermediate f^6^Am has not been reported. **(D)** The demethylation process of m^1^A remains unclear due to the special chemical bond. UTR, untranslated region; CDS, coding sequence; FTO, fat mass and obesity-associated protein; ALKBH3, AlkB homolog 3; A, adenosine; Am, 2’-O-methyladenosine; m^6^A, N^6^-methyladenosine; hm^6^A, N^6^-hydroxymethyladenosine; f^6^A, N^6^-formyladenosine; m^6^Am, 2’-O-dimethyladenosine; hm6Am, N^6^-hydroxymethyl, 2’-O-methyladenosine; m^1^A, N^1^-methyladenosine.

As reported, m^6^A modification occurs in almost all transcripts with the ratio of m^6^A/A in mRNAs ranges from 0.2 to 0.5% (15, 24, 31, 32). The distribution of m^6^A modifications are not random but strictly restricted, where they are commonly confined in the consensus sequence RRACH that refers to [G/A/U][G>A]m^6^AC[U>A>C] motif (7, 21, 33) and enriched in the long internal exons and regions next to the 3’ untranslated region (3’ UTR) within mRNAs (21, 22, 27). The deposition of m^6^A is in the introns of the precursor mRNAs (pre-mRNAs) and in primary microRNAs (pri-miRNAs), which means that the m^6^A modification can be regulated either before or simultaneously with RNAs splicing and processing (34) (**Table 1**, **Figure 1**).

**Table 1:** The biological characteristics of methyladenosine modification in mRNA.

MT	Peaks/sites	Ratio	Distribution	Motif	Detection methods
m^6^A	~10,000–20,000	~0.2–0.5% (m^6^A/A)	Introns, long internal exons, near stop codon and 3’ UTR	RRACH motif ([G/A/U][G>A]m^6^AC[U>A>C])	MeRIP-seq, SCARLET, miCLIP, SELECT, m^6^A-LAIC-seq, DART-seq, MAZTER-seq, m^6^A-REF-seq, m^6^A-lable-seq, m^6^A-SEAL, and so on
m^6^Am	~500–1,000	~0.01–0.02% (m^6^Am/A)	Cap+1/2, 5’UTR	BCm^6^Am motif (B represents C, G or U)	CITS miCLIP, refined RIP-seq, m^6^A-SEAL
m^1^A	~500–5,000 (but need more evidence)	~0.01–0.16% (m^1^A/A)	5′ UTR, near start codons or TSS	GCA codon and GUUCRA tRNA-like motif (both not obviously)	m^1^A-seq combined method, m^1^A-ID-seq, m^1^A-MAP

MT, modification types; m^6^A, N^6^-methyladenosine; m^6^Am, N^6^,2’-O-dimethyladenosine; m^1^A, N^1^-methyladenosine; A, adenosine; U, uridine; C, cytidine; G, Guanosine; 5’UTR, 5’ untranslated region; 3’UTR, 3’ untranslated region; TSS, transcription start site; MeRIP-seq, m^6^A RNA immunoprecipitation followed by high-throughput sequencing; SCARLET, site-specific cleavage and radioactive labeling followed by ligation-assisted extraction and thin layer chromatography; miCLIP, m^6^A individual-nucleotide-resolution crosslinking and immunoprecipitation; SELECT, single-base elongation and ligation-based qPCR amplification method; m6A-LAIC-seq, m^6^A level and isoform characterization sequencing; DART-seq, deamination adjacent to RNA modification targets sequencing; m^6^A-REF-seq, m^6^A-sensitive RNA-endoribonuclease-facilitated sequencing; CITS miCLIP, the crosslinking-induced truncation sites-based miCLIP.

### Components of the m^6^A Modification System

#### Writers

In 1994, Bokar and colleagues characterized a multicomponent complex of mRNA m^6^A methyltransferases (MTases, “writers”) that extracted from the nucleus, which is composed of three components with ~30 KDa, ~200 KDa, and ~875 kDa, respectively. The ~200 kDa component contains the S-adenosyl-L-methionine (SAM)-binding site on a 70 kDa subunit and the ~875kDa component may has affinity for mRNA strands (35, 36). Subsequently the SAM-binding ability of the 70 kDa subunit and was named as MT-A70 (now known as methyltransferase-like 3 (METTL3) (37). Hereafter, methyltransferase-like 14 (METTL14) has been identified as the homologue of METTL3 and functions as another core component of the complex (38–41). Both METTL3 and METTL14 are highly conserved within the (D/E)PP(W/L) active site and the SAM−binding motif in mammals with ~35 and ~43% sequence homology of the MTase domain in mouse and human respectively (39, 40, 42). Despite METTL3 and METTL14 exhibit relatively weak MTase activity when acting alone, the METTL3-METTL14 complex with a stoichiometric ratio of 1:1 shows a much higher catalytic activity. The primary functions of METTL3 in the complex is to catalyze methyl-group transfer, whereas METTL14 is the aide that helps MTase complex positioning by identifying the histone H3 trimethylation at Lys36 (H3K36me3) (in co-transcriptional manner), and offers a structural scaffold that enhancing the catalytic activity of METTL3, even though METTL14 can affect the m^6^A levels more significantly than METTL3 (40, 41, 43–46).

In addition, the Wilms tumor 1-associated protein (WTAP) that previously found to interact with the Wilms’ tumor suppressor-1 (WT1) and participates alternative pre-mRNA splicing was identified as an additional component of the m^6^A MTases complex (39, 47–49). Without the MTase domain, WTAP assists METTL3/14 to localize in the nuclear speckles where enriched with pre-mRNA processing factors and synergize to methylate the adenosines in mRNAs (39, 41).

Recent studies have identified other associated proteins in MTase complex. Schwartz et al. (50) found that KIAA1429 (VIRMA) is required for the m^6^A methylation in human cells, and Yue et al. further demonstrated that KIAA1429 can play a role as region-selective factors by recruiting the catalytic core components METTL3/METTL14/WTAP to 3’UTR and near the stop codon (51). They also highlighted the importance of Cbl proto oncogene like 1 (HAKAI or CBLL1) and zinc finger CCCH-type containing 13 (ZC3H13) in the full methylation program, and ZC3H13 is required for the nuclear localization of MTase complex (51, 52). The RNA-binding motif protein 15 (RBM15) and its paralogue RBM15B are also identified as the regulators of m^6^A modification in the lncRNA X-inactive specific transcript (XIST), as well as in mRNAs (53). In addition, the transcription factors zinc finger protein 217 (ZFP217), SMAD2/3, and CAAT-box binding protein (CEBPZ) are found to mediate the m^6^A deposition in mRNAs (54–56). Some other m^6^A methyltransferases such as METTL5, METTL16, and zinc finger CCHC-type-containing 4 (ZCCHC4) are also indispensable for m^6^A formation, especially in ncRNAs and rRNAs (57–59).

#### Erasers

FTO was recognized as the first m6A eraser (19), which was originally discovered in 1999 and was officially named in 2007 (60, 61). Bioinformatics analysis revealed that FTO is one of the non-heme Fe^II^/α-ketoglutarate(α-KG)-dependent dioxygenases (also known as non-heme Fe^II^/2-oxoglutarate (2-OG)-dependent dioxygenases) (62). FTO was shown to mediate the demethylation of N^3^-methylthymidine in single-stranded DNA and N^3^-methyluridine in single-stranded RNA *in vitro* (63, 64). In 2011, Jia et al. (19) proved that FTO could participate in the demethylation process of nuclear RNAs in nuclear speckles, and Fu et al. (65) further revealed the role of FTO in the detailed process of RNA m^6^A demethylation in 2013. They found that FTO oxidizes m^6^A in a stepwise manner, and the intermediate of N^6^-hydroxymethyladenosine (hm^6^A) is the direct oxidation product of m^6^A and turns into the form of N^6^-formyladenosine (f^6^A). The final products of m^6^A demethylation are unmethylated adenosine and formaldehyde (from hm^6^A) or formic acid (from f^6^A). Interestingly, the half-lives of hm^6^A and f^6^A are suggested to be ~3 h under physiological conditions, meaning that the decomposing of hm^6^A and f^6^A do not occur simultaneously with the oxidation of m^6^A.

As for ALKBH5, the second m^6^A demethylase identified so far in mammals, belongs to the AlkB family, a class of the non-heme Fe^II^/α-ketoglutarate (α-KG)-dependent dioxygenases superfamily which was originally shown to revert DNA base damage by catalyzed oxidative demethylation of N-alkylated nucleic acid bases (20, 66–68). Structure analysis indicates that ALKBH5 has comparable catalytic activity with FTO, whereas AlkB has low level (~17%) of the amino acid sequence identity to FTO (62, 69). While FTO can demethylate on both single-stranded RNA/DNA and double-stranded RNA/DNA (albeit low) (19, 70), ALKBH5 only demethylate the single-stranded RNA/DNA with the sequence preference (the activity in the consensus sequence is twice that in other sequences), which may be due to the fact that ALKBH5 mainly localizes in nuclear speckles and acts in regulating the nuclear export and metabolism of RNAs (20).

#### Readers

There are four main readers selectively bind the m^6^A-containing mRNAs in the nucleus. In 1998 Imai et al. (71) isolated a novel RNA splicing-related protein YT521 by using yeast two-hybrid screens system with rat transformer-2-beta1 (RA301) as bait, and Hartmann et al. identified a homologous protein YT521-B by using htra2-beta1 as bait (72). Subsequently, the YT521-B homology (YTH) domain was defined as a new protein family, the YTH (YT521-B homology) domain containing protein family, and now YT521-B is known as the YTH domain-containing protein 1 (YTHDC1) (73). YTHDC1 localizes in a subnuclear structure named YT bodies that contain transcriptionally active sites and are close to other subnuclear compartments such as speckles and coiled bodies (74). Structure analysis demonstrated that the GG(m^6^A)C sequence is the preferred binding site for YTH domain in YTHDC1 (75, 76). Since its localization is adjacent to the nuclear speckles, YTHDC1 is found to participate in pre-mRNAs splicing containing m^6^A sites, and mediate its nuclear export. YTHDC1 facilitates the splicing pattern of exon inclusion in targeted mRNAs by recruiting pre-mRNA splicing factor SRSF3 (SRp20) and inhibiting SRSF10 (SRp38), by which it changes alternative splicing patterns *via* modulating splice sites selection in a concentration-dependent manner (72, 77). In addition, YTHDC1 is found to interact with the nuclear RNA export factor 1 (NXF1) to promote the nuclear export of the m^6^A-containing mRNAs (78).

The other three readers in the nucleus belong to the heterogeneous nuclear ribonucleoprotein (hnRNP) family that is composed of more than 20 members. HnRNP protein contains at least one RNA-binding domain with RNA recognition motif (RRM), K-Homology (KH) domain, or an arginine/glycine-rich box (79). Recently, their role as the “reader” of m^6^A remains controversial. Previously, Alarcon et al. showed that hnRNPA2/B1 can directly bind with m^6^A by matching the m^6^A consensus motif and regulate the alternative splicing of its mRNA targets (80), whereas Wu et al. (81) suggested that hnRNPA2/B1 may interact with m^6^A *via* the “m^6^A switch” mechanism instead of directly recognizing the m^6^A-containing bases, by which the m^6^A controls the RNA-structure-dependent accessibility of the RNA-binding domains to affect the RNA-protein interactions for biological regulation. In addition, heterogeneous nuclear ribonucleoprotein C (hnRNPC), an abundant nuclear RNA-binding protein, and heterogeneous nuclear ribonucleoprotein G (hnRNPG), a low-complexity protein, interact with the m^6^A-containing mRNAs *via* the similar “m^6^A switch” mechanism (6, 82, 83).

Besides readers in the nucleus, four members of the YTH domain-containing proteins are identified in the cytoplasm that are involved in mRNAs metabolism *via* interacting the m^6^A with their hydrophobic pocket, an aromatic cage formed by tryptophan residues, within the YTH domain (76, 84, 85). YTHDF1, YTHDF2, and YTHDF3 are highly homologous, and all contain a ~40 kDa low-complexity domain and a prion-like domain (86). The most abundant YTHDF paralog, YTHDF2, is the first member to be fully studied, where it was originally implicated in regulating the instability and the decay of the m^6^A-containing mRNAs by localizing the complex of YTHDF2-m^6^A-mRNA from the translatable pool to the processing bodies (P-bodies) (87). However, another group demonstrated that the P-bodies only act an indirect role in the decay of m^6^A-containing RNAs since no direct interaction between YTHDF2 and GW182, the core component of the P-bodies was found (88). Subsequent study revealed that YTHDF2-m^6^A-mRNA complex was located in the stress granules or neuronal RNA granules through the phase separation mechanism upon stress stimulation and was subject to compartment-specific regulations (89).

**Figure 2:**
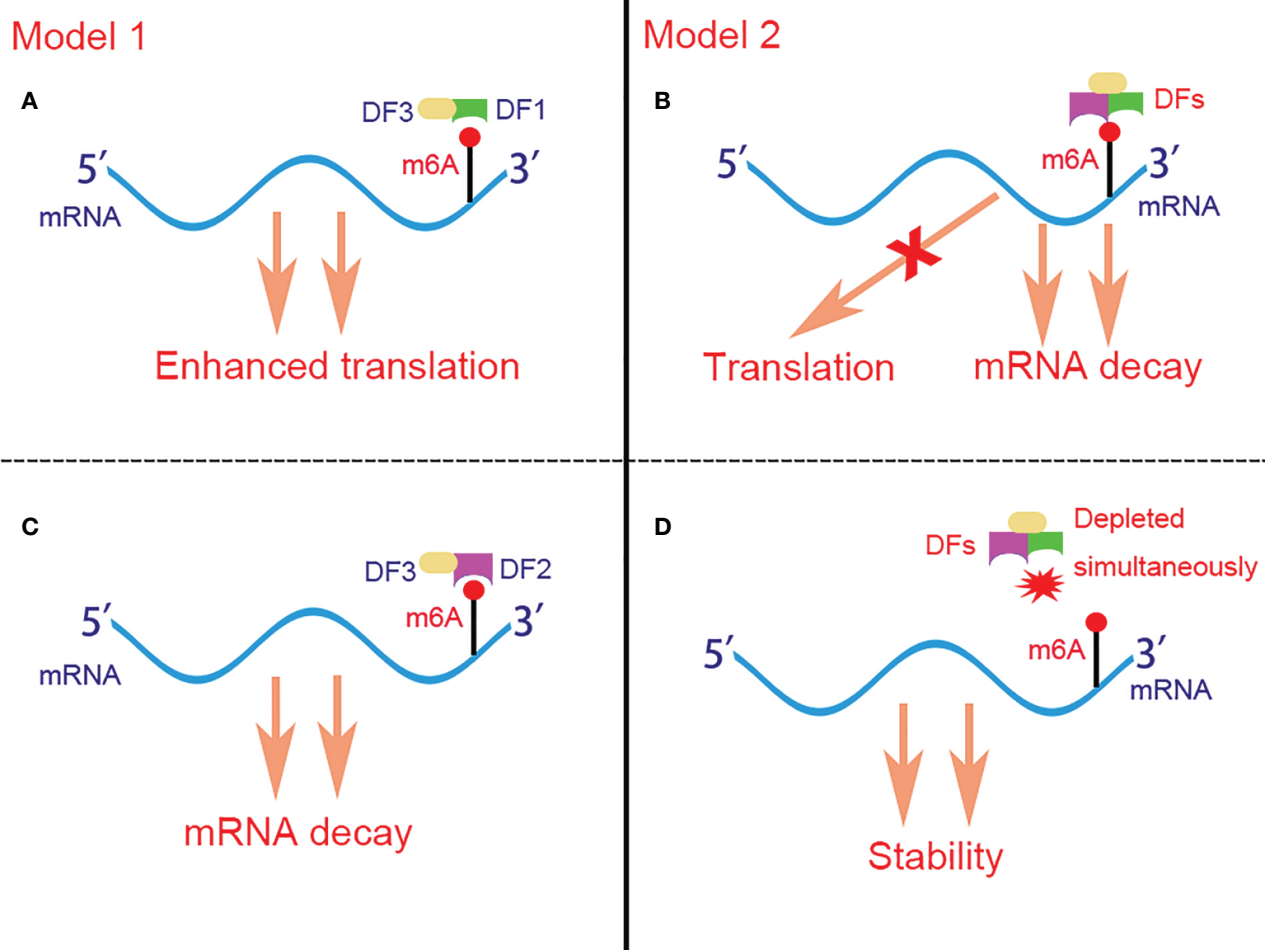
Two controversial models for the function of DFs in regulating m^6^A-containing mRNAs. In model 1 **(A, C)**, DF1 and DF2 bind to different mRNAs and promote their translation and degradation respectively. In model 2 **(B, D)**, DFs bind to the same mRNA rather than different mRNAs simultaneously and act redundantly to co-mediate mRNA degradation but not translation. The stability of mRNA can be restored only when DF1-3 are depleted simultaneously. DFs, YTHDF proteins; DF1, YTHDF1; DF2, YTHDF2; DF3, YTHDF3; m^6^A, N^6^-methyladenosine.

Although all YTHDF proteins can recruit CCR4-NOT and promote mRNA deadenylation (88), YTHDF1 is the only member which is reported to facilitate translation by binding at the RRACH motifs instead of the flanking sequence that cluster around the stop codon and subsequently recruiting translation initiation factor (eIF) and ribosome. The association of YTHDF1 with translational initiation machinery may be depend on the loop structure mediated by eIF4G and the interaction of YTHDF1 with eIF3 (90). Besides, YTHDF1 is found to bind to the nascent methylated mRNAs earlier than YTHDF2, which suggests that the translation of mRNAs occurs before their decay under various physiological conditions (90). As for YTHDF3, it plays dual functions in m^6^A-containing mRNAs metabolism by either promoting the translation of the targeted mRNAs *via* interaction with YTHDF1 (91), or accelerating the decay of the targeted mRNAs *via* interaction with YTHDF2 (92). Controversially, Jaffrey et al. (93) proposed another brand new but opposite model for the role of YTHDF proteins in regulating m^6^A-containing mRNAs. They demonstrated that YTHDF proteins binded with the same mRNA rather than different mRNAs and act redundantly to co-mediate mRNA degradation, and the stability of mRNA fails to restore until all YTHDF1,2,3 are depleted simultaneously (**Figure 2**).

The fifth member of the YTH protein family is YTHDC2, which is different from the other cytoplasmic “readers”. YTHDC2 has a large molecular mass of ~160 kDa and contains the helicase domain (94). YTHDC2 is previously reported to enhance the translation efficiency of its targets and decrease their mRNA abundance by binding to the m^6^A site at its consensus motif and influencing the mRNA secondary structures (94, 95). However, a latest report indicated that YTHDC2 could also reduce the m^6^A-containing mRNAs stability and inhibit gene expression in certain situations (96).

The insulin-like growth factor 2 mRNA-binding proteins (IGF2BPs, originally called IMPs) family, including IGF2BP1/2/3, which is initially recognized as an IGF2 translation inhibitor (97), belongs to a new family of m^6^A readers that mainly prevent the m^6^A-containing mRNAs from degradation in cytoplasm (98). IGF2BPs are composed of two RRM domains and four KH domains, and preferentially bind the m^6^A-modified mRNAs through recognizing the consensus GG(m^6^A)C sequence and facilitate the stability and translation of thousands of its mRNA targets by co-localizing in the P-bodies or stress granules, thus upregulating the gene expression in globally (98). Recently, ELAV like RNA binding protein 1 (ELAVL1, also known as HuR), matrin 3 (MATR3), and poly (A) binding protein cytoplasmic 1 (PABPC1) have been identified as the cofactors of IGF2BPs that promote the stability of m^6^A-containing mRNAs simultaneously.

In addition, fragile X mental retardation protein (FMRP) and proline rich coiled-coil 2 A (PRRC2A) are reported to play a role as the reader/stabilizer of the m^6^A-containing mRNAs (99, 100). METTL3 is found to associate with ribosomes and promote translation in some cancers when it localizes in the cytoplasm (101, 102). Moreover, accumulated readers of the m^6^A in ncRNAs is summarized in **Table 2**.

**Table 2:** The main components of methyladenosine modification systems^*^.

MT	Writers	Erasers	Readers and its functions			
m^6^A	METTL3/14, WTAP, KIAA1429 (VIRMA), RBM15/15B, ZC3H13, CBLL1, ZFP217, SMAD2/3, CEBPZ, METTL5, METTL16, ZCCHC4	FTO, ALKBH5	In nucleus	YTHDC1		Mediating splicing and nuclear export
				hnRNPA2/B1, hnRNPC and hnRNPG		Mediating splicing
			In cytoplasm	YTHDC2		Diversify
				YTHDF1 and METTL3		Facilitating translation
				YTHDF2		Facilitating decay
				YTHDF3		Work with YTHDF1 or YTHDF2
				IGF2BP1/2/3, FMRP, PRRC2A		Facilitating stability
m^6^Am	PCIF1 (CAPAM), METTL4	FTO	There is no m^6^Am reader that has been identified; and the functions of it still controversial (facilitating or preventing decay and translation)?			
m^1^A	TRMT10C, TRMT6/61A, TRMT61B, BMT2, NML	FTO, ALKBH1, ALKBH3	YTHDF1		Facilitating translation	
			YTHDF2/3		Promoting decay	
			YTHDC1		Unclear	

MT, modification types; writers, methyltransferases; erasers, demethylases; readers, the proteins that bind to methylation modifications; m^6^A, N^6^-methyladenosine; m^6^Am, N^6^,2’-O-dimethyladenosine; m^1^A, N^1^-methyladenosine; METTL3/5/14/16, methyltransferase-like3/5/14/15; WTAP, Wilms tumor 1-associated protein; RBM15/15B, RNA-binding motif protein 15/15B; ZC3H13, zinc finger CCCH-type containing 13; CBLL1, Cbl photo oncogene like 1; ZFP217, zinc finger protein 217; CEBPZ, CAATT-box binding protein; ZCCHC4, CCHC-type-containing 4; FTO, fat mass and obesity-associated protein; ALKBH1/3/5, AlkB homolog 1/3/5; YTHDC1/2, YT521-B homology (YTH) domain-containing protein 1/2; hnRNPA2/B1, heterogeneous nuclear ribonucleoprotein A2/B1; hnRNPC, heterogeneous nuclear ribonucleoprotein C; hnRNPG, heterogeneous nuclear ribonucleoprotein G; FMRP, fragile X mental retardation protein; PRRC2A, proline rich coiled-coil 2 A; PCIF1, phosphorylated CTD-interacting factor 1; TRMT10C/6/61A/61B, tRNA methyltransferase 10C/6/61A/61B; BMT2, base methyltransferase of 25S RNA; NML, nucleomethylin. *Has been identified at present.

## The m^6^Am Modification

### Biological Characteristics of m^6^Am Modification

The m^6^Am is the second most prevalent modification in cellular mRNAs and in some small nuclear RNAs (snRNAs). The structure of mRNAs after the m^7^G cap can be divided into three main types, the m^7^G5’ppp5’NmpNp (p denotes phosphate group, Nm and N denote 2’-O-methylated nucleotide and nucleotide respectively), the m^7^G5’ppp5’NpNp and the m^7^G5’ppp5’NmpNmpNp (15, 103, 104). Recently, the 2’-O-methyladenosine (Am) was showed to be the first nucleotide adjacent to the m^7^G cap and it can be further modified at the N^6^ position by methylation to generate m^6^Am (92% chance of being modified), where the structure of m^7^G5’ppp5’m^6^AmpNp comprises 20–30% of all the structures (105, 106) (**Figure 1**). The second nucleotide can harbor a similar modification but with a lower frequency, whereas m^6^Am rarely located in the third nucleotide and m^6^A or A has not been found at the first nucleotide position (107). In addition, there are ~6% of the m^6^Am occurs outside the 5’UTR, and motif analysis reveals that the m^6^Am mainly deposit in a novel motif BCm^6^Am (B represents C, G, or U) that enriched in the transcription start site (TSS), rather than the canonical m^6^A motif RRACH (4, 108, 109). Molinie et al. used liquid chromatography-mass spectrometry (LC-MS) to quantify of the m^6^Am in mRNAs and found that mRNAs contain ~3 m^6^Am nucleotides per 10^5^ nucleotides, revealing a 33-fold level of the m^6^A modification than the m^6^Am in mRNAs (24). Consistently, Liu et al. (108) confirmed the m^6^Am/A ratio of total RNAs and mRNAs ranges from 0.0036 to 0.0169% and from ~0.01 to 0.02% respectively. Currently, the m^6^Am transcriptome-wide expression can only be detected by the methods of the crosslinking-induced truncation sites-based miCLIP (CITS miCLIP) and the refined RIP-seq, and an antibody-free enzyme-assisted chemical approach termed m^6^A-SEAL (29) (**Table 1**).

### Components of m^6^Am Modification System

#### Writer

The formation of m^6^Am occurs on the basis of Am that formed by the MTases HENMT1 and FTSJ3 (110, 111), and its modification system rarely known yet. The m^6^Am MTase was previously purified with a molecular weight of ~65 KD in 1978, and phosphorylated CTD-interacting factor 1 (PCIF1) was recognized as the first m^6^Am MTase in 2019. It was named by its ability to directly bind to the phosphorylated carboxyl-terminal domain (CTD) of RNA polymerase II (RNAP II) by its WW domain, also called cap-specific adenosine methyltransferase (CAPAM) (106, 112, 113). Unlike the m^6^A core readers that work in the form of a methyltransferase complex, PCIF1 is a “stand-alone” RNA MTase and functions in an m^7^G cap-dependent manner. Recently, METTL4 was reported to catalyze m^6^Am methylation in the U2 snRNA (114).

### Eraser

FTO, the first m^6^A demethylase, was found to mediate the demethylation of m^6^Am in the similar manner to that of m^6^A (115, 116). The intermediate of N^6^-hydroxymethyl, 2’-O-methyladenosine (hm^6^Am) was detected as well as the end product Am. Intriguingly, although both m^6^A and m^6^Am can be catalyzed by FTO, the priority between them is still controversial. Zhang et al. (116) showed that FTO displays the similar demethylation activity toward internal m^6^A and cap m^6^Am modifications *in vivo* and *in vitro*. But He et al. revealed that FTO shows different affinity to the m^6^A and the m^6^Am among cells where the m^6^A is most affected despite it prefers the m^6^Am *in vitro* by the cellular cap-binding proteins (117). Controversially, Mauer et al. (115) found that FTO does not efficiently demethylate m^6^A but preferentially demethylates m^6^Am. They further showed that ALKBH5 did not affect the m^6^Am in mRNAs and stated that FTO may targets the m^6^Am whereas ALKBH5 targets the m^6^A *in vivo*.

### Functions of the m^6^Am

Currently, there is no m^6^Am reader that has been identified, and its function remains controversial. The first work showed that the m^6^Am stabilizes mRNA by preventing the mRNA-decapping enzyme DCP2-mediated decapping and microRNA-mediated mRNA degradation (115), and it was confirmed by Mauer’s work (109). However, Sendinc et al. (118) revealed that m^6^Am fails to alter mRNA transcription and stability, and negatively impacts cap-dependent translation. Akichika et al. (106) further showed that the m^6^Am facilitates the translation of capped mRNAs. The direct readers of the m^6^Am are under investigated (**Table 2**).

## The m^1^A Modification

### Biological Characteristics of the m^1^A Modification

The m^1^A modification was firstly discovered in the total mixed RNA samples in 1961 (119) and was found that it can rearrange into the m^6^A under alkaline conditions (Dimroth rearrangement) in 1968 (120). Subsequently, accumulating evidence has shown that the m^1^A occurs in rRNAs and tRNAs where the m^1^A is typically found at position 9 and 58 in the tRNA TΨC-loop and plays key roles in the structure formation and function execution *via* its methyl adduct and positive charge (121, 122). By using of the liquid chromatography-tandem mass spectrometry (LC-MS/MS), the ratio of m^1^A/A in the mammalian cell lines and tissues can be easily detected, which ranging from approximately 0.015 to 0.054% and up to 0.16% (123, 124). Hereafter, He et al. (123) used the combined method of an antibody-based approach called m^1^A-seq and an orthogonal chemical method based on Dimroth rearrangement to obtain a more detailed distribution of m^1^A. They found that the distribution pattern and the peaks of the m^1^A are highly conserved in the samples from multiple sources, and the m^1^A enrich in the 5′ UTR, near the start codons or TSS, which is similar to that of m^6^Am. Yi et al. further supported the finding by original technology m^1^A-ID-seq (124). In addition, single-nucleotide resolution analysis (m^1^A-MAP) showed that the m^1^A lacks of obvious preference to certain motif, but the GCA codon and GUUCRA tRNA-like motif are frequently modified, and no m^1^A is detected in the AUG start codon (123, 125). Finally, Safra et al. reported 15 m^1^A sites in mRNAs and lncRNAs (126) (**Table 1**) (**Figure 1**).

### Components of m^1^A Modification System

#### Writers

Although a variety of the m^1^A MTases, including tRNA methyltransferase 10C (TRMT10C), TRMT6/61A, TRMT61B, base MTase of 25S RNA (BMT2), MTR1, and nucleomethylin (NML), have been discovered, most of them catalyze the sites on tRNAs or rRNAs (122, 127–130). Li et al. unveiled that TRMT6/61A is able to methylate the m^1^A sites that are confined in GUUCRA tRNA-like motifs in mRNAs, and some of the mitochondrial (mt)-mRNAs are the target of TRMT61B (125). In addition, Safra et al. (126) have identified that a single m^1^A site in the mt-ND5 mRNA which is catalyzed by TRMT10C. Nonetheless, there is no direct specific m^1^A writer has been identified for mRNA yet.

#### Erasers

The m^1^A demethylases are found to only catalyze tRNAs so far. He et al. (131) showed that the human homolog of *E. coli* AlkB ALKBH1 is an important eraser that catalyzes the demethylation of the m^1^A in tRNAs in 2016, and FTO, was proven to mediate the m^1^A demethylation in tRNAs (117). However, neither ALKBH1 nor FTO mediates the removal of the methyl group from m^1^A in mRNAs. Recently, another demethylase ALKBH3 was shown to have a strong preference for single stranded DNA/RNA and the ability of repairing methylation damage to RNA *in vitro* in both tRNAs and mRNAs (123, 124, 132, 133). Yi et al. (124) further showed that ALKBH3 has minimal sequence preference and acts globally in the transcriptome.

### Functions of m^1^A

The process of eukaryotic protein translation, especially the initiation step of translation, is strictly regulated in cells. Structure analysis showed that the secondary structure in the 5’UTR which is the target of the initiation factors such as eIF4A/B/H complex can affect the efficiency of the initiation of translation and the early elongation by impeding the binding and movement of the 40S ribosome (134, 135). He et al. (123) suggested that the m^1^A plays a positive role for the translation initiation in mammalian mRNAs, which is further supported by Li et al. (125). Mechanically, the m^1^A may inhibit Watson-Crick base pairing or introduce charge-charge interactions, leading to the alteration of the secondary/tertiary structure of 5’UTR in mRNAs. Potential readers specifically bound to the m^1^A in mRNAs are supposed to promote the initiation of translation, which is analogous to the role of YTHDF1 in translation enhancement. However, there are controversial reports showed that the m^1^A can repress the translation of mRNAs, especially mt-mRNAs while the underlying mechanism remains to be explored (126). In addition, the m^1^A is found to promote mRNA degradation by interacting with its potential readers YTHDF2/3 (136, 137) (**Table 2**).

## Links with Gastrointestinal Cancers and Potential Therapeutic Strategies

Under normal physiological condition, methylation modification is precisely modulated by the methyltransferases and demethylases, and involved in regulating alternative splicing, nuclear export, stability, translation, or degradation of the methylated RNAs, thereby affecting cell self-renew, cell proliferation, and cell differentiation. Recently, accumulating studies have revealed that abnormality in RNA methylation leaded by mutations or dysregulation that cause the gain or the loss of methylation sites are closely related to the initiation, progression metastasis, and suppression of various tumors including GICs (138).

### Aberrant Writers in Gastrointestinal-Related Cancers

The writer, METTL3, is found to be upregulated in GC patients with poor prognosis, which is caused by the P300-mediated H3K27 acetylation activation in the promoter of METTL3 and mediation by the transcription factor GFI1 (139–143). Yue et al. (139) have identified the zinc finger MYM-type containing 1 (ZMYM1) mRNA as the direct target of METTL3. Mechanistically, the reader ELAVL1 binds to the m^6^A sites within ZMYM1 mRNA and enhances the stability of ZMYM1. The induced ZMYM1 further inhibits the expression of E-cadherin by forming a complex of CtBP/LSD1/CoREST/ZMYM1 in the promoter region of E-cadherin, thus stimulating the epithelial-mesenchymal transition (EMT) and promoting metastasis of GC. In another report, the m^6^A modification of hepatoma derived growth factor (HDGF) mRNA can be induced by high level of METTL3, and recognized by the reader IGF2BP3 to promote its stability. The upregulated HDGF could further facilitate tumor angiogenesis and increase glycolysis in GC, which in turn enhance the tumor growth and liver metastasis (140). Additionally, the mRNAs of pre-protein translocation factor (SEC62), ARHGAP5, and MCM5 and MCM6 (the component molecules in the MYC pathway) are highly modified by the aberrant METTL3, and led to the acceleration of GC progression (141, 143, 144).

Upregulated METTL3 in CRC primary or metastatic tissues is highly associated with unfavorable outcomes (145–150). One potential mechanism mediated by upregulated METTL3 is ceramide glycosylation that generates glycosphingolipids (particularly globotriaosylceramide) and activates cSrc and β-catenin signaling (151). Li et al. (145) unveiled that higher METTL3 expression in CRC facilitates the methylation of SRY (sex determining region Y)-box 2 (SOX2) mRNA, and the reader IGF2BP2 further recognized the m^6^A-containing SOX2 mRNA and induced the expression level of SOX2 protein. SOX2 was previously reported to control the properties of the stem cells and enhance cell proliferation and invasion in squamous cell carcinoma (152). While in CRC, highly expressed SOX2 regulated its downstream targets, including *cyclin D1* (*CCND1*), *MYC (*mainly referred to as *c-Myc)*, and *POU class 5 homeobox 1* (*POU5F1*), and promoted their expression levels, thus upregulating CD133, CD44, and epithelial cell adhesion molecule (EpCAM). Shen et al. (148) found that METTL3 can directly interacts with the 5’/3’UTR regions of Hexokinase 2 (HK2) mRNA and the 3’UTR region of Glucose transporter type 1 (GLUT1, also SLC2A1) mRNA, and subsequently stabilized their mRNAs and activated the glycolysis pathway in CRC cells in a IGF2BP2- or IGF2BP2/3-dependent manner. In addition, upregulated METTL3 could facilitate CRC cell proliferation, progression, and metastasis by various signaling pathways including miR-1246/SPRED2/MAPK signaling pathway, p38/ERK pathway, and cyclin E1 (CCNE1) cell proliferation pathway (147, 149, 150).

Analogously, the high level of other writers WTAP and RBM15 also predicts poor prognosis for GC (153–155) Li et al. found that WTAP could be served as an independent predictor of GC and its high expression is closely related to the low T lymphocyte infiltration and T cell-related immune response (154).

Intriguingly, the writer METTL14 is reported to be downregulated in GC and CRC patients (139, 156). Zhang et al. (156) unveiled that METTL14 suppression may cause activation of the Wnt and PI3K-Akt signaling and thus promote GC progression. While Yang et al. (157) have revealed that the downregulated METTL14 is associated with the poor outcomes of CRC patients through up-regulating oncogenic lncRNA XIST. Specifically, the m^6^A level within lncRNA XIST is reduced as METTL14 suppression, which could lead to the RNA degradation and decay mediated by the m^6^A reader YTHDF2. The abundant lncRNA XIST due to downregulation of METTL14 acts as a carcinogen and promote cell proliferation and metastasis in CRC (158). Additionally, the downregulated METTL14 affects the m^6^A level in pri-miR-375, by which it decreased the binding of DGCR8 to pri-miR-375 and results in the reduction of mature miR-375. The reduction of miR-375 causes induced level of Yes-associated protein 1 (YAP1) and SP1, and ultimately leads to cell growth in CRC *via* miR-375/YAP1 pathway and cell invasion *via* miR-375/SP1 pathway (159).

### Aberrant Erasers in Gastrointestinal-Related Cancers

FTO, the first mammalian m^6^A demethylase and the only m^6^Am demethylase currently discovered, is found to mediate the progression in GICs. FTO was reported to serve as an independent prognostic marker due to its frequently higher expression in high-risk scores subtype of GC (153, 155, 160). Other erasers ALKBH3 and ALKBH5 are also upregulated in GC, and ALKBH5 is found to promote the invasion and metastasis of GC by interacting with the lncRNA NEAT1 (nuclear paraspeckle assembly transcript 1) (155, 161, 162).

However, the expression levels of ALKBH5 and FTO in CRC are still controversial (146). From The Cancer Genome Atlas (TCGA), the Gene Expression Omnibus (GEO) database, and the Human Protein Atlas, ALKBH5 shows weak expression in CRC tissues compared to the normal tissues, and FTO shows no significant difference between CRC tissues and normal tissues. Whereas Wu et al. revealed a potential CRC-promoting mechanism *via* the ALKBH5/m^6^A/RP11/hnRNPA2B1/E-ligases/Zeb1 axis (163). They found that lncRNA RP11 in CRC is highly expressed and associated with the CRC stage in patients, by which lncRNA RP11 is regulated in an m^6^A-dependent manner and negatively correlated with ALKBH5 although METTL3 is elevated in CRC patients. Mechanistically, m^6^A-containing RP11 can interact with the reader hnRNPA2B1 and bind to its downstream targets, two E3-ligase mRNAs Siah1 and Fbxo45 to accelerate their decay. The reduced Siah1 and Fbxo45 further downregulates the EMT-transcription factors Zeb1, and ultimately leads to the development of CRC. In addition, Relier et al. (164) showed that low expression of FTO in CRC cells causes increase of the m^6^Am levels in mRNAs and results in the enhanced malignancy and chemo resistance in CRC cells, which can be partially reversed by inhibition of PCIF1.

### Aberrant Readers in Gastrointestinal-Related Cancers

Emerging studies have reported the upstream regulatory mechanisms that lead to generation of the aberrant readers in CRC. Wang et al. (165) have identified a novel lncRNA LINRIS to stabilize IGF2BP2 *via* LINRIS/IGF2BP2/MYC axis and promote cell proliferation in CRC. Mechanistically, the elevated level of LINRIS in the CRC patients with unfavorable prognostic could act on IGF2BP2 and protect it from K139 ubiquitination and autophagy degradation, and maintain its stability. The upregulated IGF2BP2 subsequently promotes the expression of its downstream target MYC mRNA, and enhances the MYC-mediated glycolysis in CRC, which eventually leads to progression of CRC. Inhibition of this axis by GATA3 may provide a potential therapeutic strategy for CRC.

Recently, Ni et al. showed another lncRNA involved in the YAP signaling pathway during CRC progression *via* the GAS5/YAP/YTHDF3 axis (166). They found GAS5 is downregulated in most of CRC tissues and is negatively correlated with the protein levels of YAP and YTHDF3, while the increased YTHDF3 is a significant prognostic factor for poor overall survival in CRC patients (146). Mechanistically, downregulation of GAS5 inhibits phosphorylation of YAP and attenuates its ubiquitination and degradation. The increased YAP further promotes expression level of YTHDF3, however, the downstream regulatory pathway of YTHDF3 that facilitates CRC progression is unclear.

Additionally, YTHDF1 is reported to be overexpressed in CRC and plays a vital oncogenic role in CRC (146, 167). Silencing YTHDF1 not only reduces the number of colon spheres but also causes significant downregulation of cancer stem cell markers, including CD44, CD133, OCT4, ALDH1, and Lgr5 in CRC cells. These findings indicate that YTHDF1 plays a key role in maintaining CRC stemness, which is analogous to the role of METTL3 in CRC (145). YTHDF1 is found to regulate the Wnt/β-Catenin pathway in CRC as well (167). Silencing YTHDF1 leads to reduction of the expressions of the nonphospho (active)-β-catenin and the Wnt/β-catenin downstream targets, including c-JUN, CCND1, and CD44, and thus downregulates the β-catenin nuclear signals activity.

### Other Gastric Cancer/Colorectal Cancer-Promoting Mechanisms Related to Methylation

Recent study has revealed that the m^6^A modification in a circular RNA (circRNA), circNSUN2 that maps to the chromosome 5p15 amplicon in CRC, has an important role for promoting CRC liver metastasis (168). Mechanically, circNSUN2 contains an m^6^A motif within its exon 5-exon 4 junction sequence where it can be modified by METTL3, and then YTHDC1 facilitates the nuclear export of m^6^A-containing circNSUN2. In cytoplasm, circNSUN2 stabilizes its downstream target HMGA2 mRNA by forming the complex with an m^6^A reader IGF2BP2, and promoting the EMT process and liver metastasis in CRC. Interestingly, upregulation of circNSUN2 in CRC is in an METTL3-independent manner, and silencing METTL3 does not change total expression of circNSUN2 nor increases the nuclear content or reduces the cytoplasmic content.

Mutations that cause the gain or loss of methylation sites are found to involve in generation, progression and drug resistance of GICs. Uddin et al. (151) have shown that the point-mutated codon 273 (G > A) of p53 pre-mRNA promotes expression of mutant protein in a METTL3- and m^6^A-dependent manner, while the upregulated METTL3 is partly caused by a serial ceramide glycosylation mechanism. The mutant protein is found to lose its original role of cancer suppressing and obtain many oncogenic functions to generate the acquired multidrug resistance in CRC. Further, they showed that either silencing METTL3 by small interfering RNA (siRNA), or inhibiting RNA methylation with neplanocin A, or suppressing ceramide glycosylation is able to re-sensitize the resistant CRC cells to anticancer drugs. Recently, Tian et al. (169) revealed another type of mutations that are related to the m^6^A modification, the missense variant rs8100241 (G > A) located in ANKLE1. Overexpression of the rs8100241[A] allele significantly increased the ANKLE1 m^6^A level that was catalyzed by writers METTL3/14 and WTAP and recognized by reader of YTHDF1, thus the dysregulated ANKLE1 protein is facilitated compared to that of rs8100241[G] allele, which is significantly related to susceptibility of CRC.

## Potential Therapeutic Strategies

**Figure 3:**
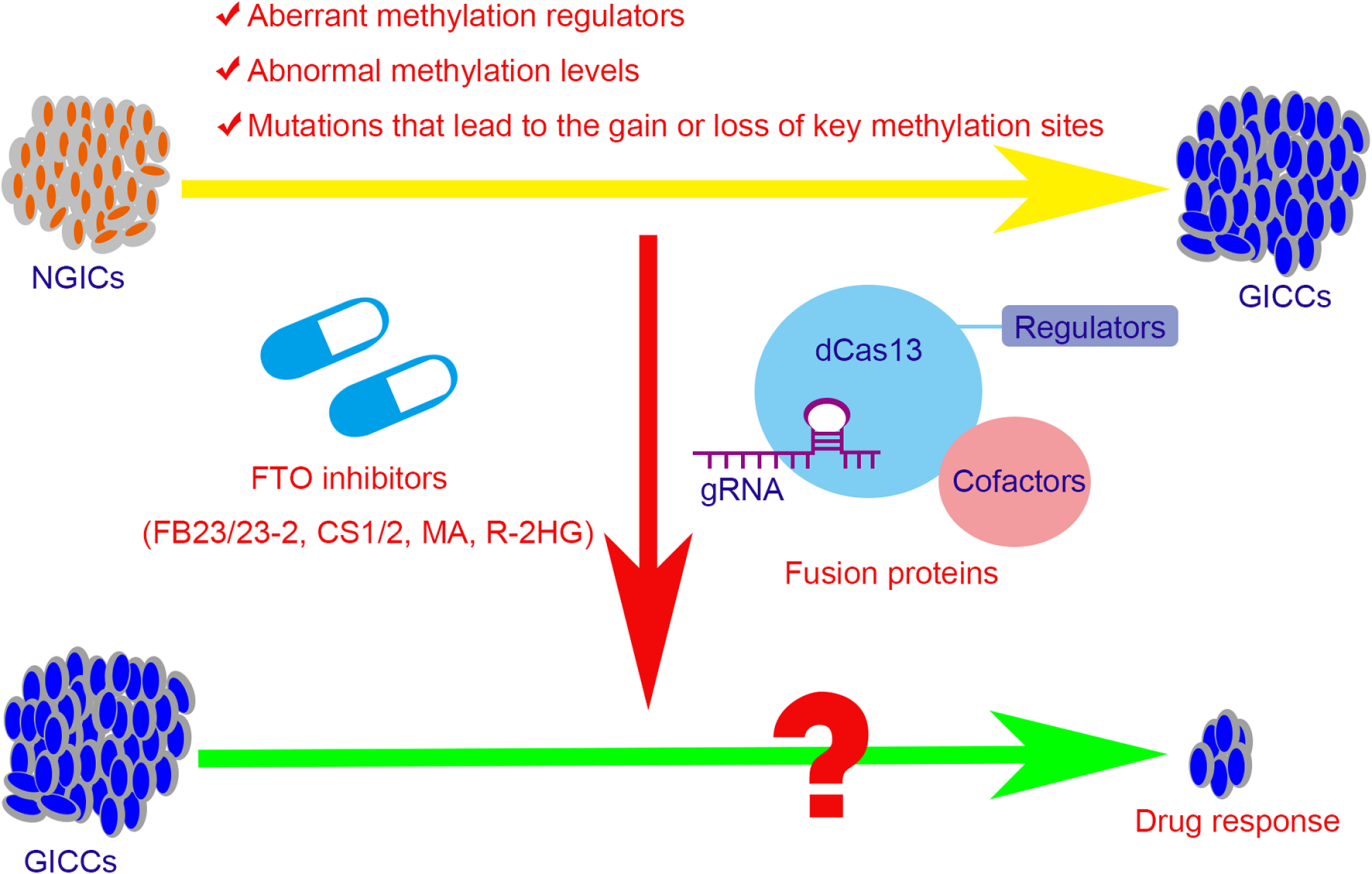
The potential therapeutic strategies for the GICs with abnormal methylation regulators or levels. The aberrant methylation regulators, abnormal methylation levels, and the mutations that lead to the gain or loss of key methylation sites contribute partly in tumorigenesis, progression, and drug resistance in GICs. The potential therapeutic strategies include the small molecule inhibitors of the regulators and the targeted fusion proteins that based on CRISPR/Cas 13 system. FTO, fat mass and obesity-associated protein; NGICs, normal gastrointestinal cells; GICCs, gastrointestinal cancer cells; gRNA, guide RNA; dCas13, inactive Cas13 enzyme; MA, meclofenamic acid; R-2HG, R-2-hydroxyglutarate.

In view of the relationship between methylation modifications and tumors, new tumor treatment strategies have been explored. Meclofenamic acid (MA), the non-steroidal anti-inflammatory drug, was found to compete with FTO binding for the m^6^A-containing nucleic acid and functions as FTO inhibitor (170). R-2-hydroxyglutarate (R-2HG), generated from mutant isocitrate dehydrogenase 1/2 (IDH1/2) enzymes, was also found to inhibit FTO activity and increase the m^6^A level in cells, which in turn decreases the stability of MYC/CEBPA and thus block the MYC pathways (171). Recently, two synthetic high-efficient FTO inhibitors are identified. Chen et al. (172) have developed two potent FTO inhibitors FB23 and FB23-2 and showed that they could directly bind to FTO and selectively block the m^6^A demethylase activity of FTO. Subsequently, they further developed two others promising FTO inhibitors, namely CS1 and CS2, which exhibit strong anti-tumor effects in multiple types of cancers. For leukemia cells, FTO inhibitors can not only block the signal axis of FTO/m^6^A/MYC/CEBPA and inhibit the self-renewal of cancer stem cells, but also suppress the expression of immune checkpoint LILRB4 and immune evasion thus enhancing the cytotoxicity of T cells (173). However, the inhibitors of other m^6^A regulators such as METTL3, METTL14, or WTAP have not been systematically developed. Moreover, targeted RNA demethylation or methylation by the engineered dCas13-containing fusion proteins may hold the potential to develop a treatment regimen for GICs (174–176) (**Figure 3**).

## Conclusions and Future Perspective

Since 2011, extensive studies have worked on the methylation modifications in RNAs, providing an extensive and accumulating database including m^6^A, m^6^Am, and m^1^A. The formation of m^1^A, m^6^A, and m^6^Am is no substantial correlated, and the roles of m^6^Am and m^1^A are partly similar to that of m^6^A, by which the m^6^Am and m^6^A modifications are demethylated by FTO. It would be interesting to measure the interference produced by m^6^Am and m^1^A during the m^6^A exploration process.

The dysregulation of RNA methylation has been linked to the abnormalities in the MYC pathway, the Wnt/β-Catenin pathway, the ErbB2 pathway, the PI3K-AKT pathway and EMT in many human cancers. For GICs, the upregulated METTL3 and erasers are mainly involved in the MAPK/ERK pathway, the CCNE1 pathway, the SOX2/tumor stemness pathway, glycolipid metabolism, and EMT and to facilitate CRC formation and progression, whereas the low expression of METTL14 mediates the lncRNA XIST axis and the miR-375/YAP1 and SP1 axis to promote CRC progression. Moreover, the upregulated readers, IGF2BP2, YTHDF3, and YTHDF1, represent as poor prognosis factors in CRC by regulating the lncRNA LINRIS/IGF2BP2/MYC/glycolysis axis, the lncRNA GAS5/YAP/YTHDF3 axis and the YTHDF1/Wnt/β-Catenin and tumor stemness pathway respectively (**Table 3**). Mutations that cause the gain of methylation sites, including the point-mutated codon 273 (G > A) of p53 pre-mRNA and the missense variant rs8100241 (G > A) located in ANKLE1, are also linked to tumorigenesis, progression and drug resistance in CRC.

**Table 3:** Relationship between aberrant regulators and GICs*.

Cancer types	Regulator	Role in RNA modification	Abnormal change	Results	Mechanisms/targets	Ref
GC	METTL3	Writer	Upregulated	Poor prognosis	METTL3/m^6^A/ZMYM1/ELAVL1/E-cadherin/EMT axis	(139)
					METTL3/m^6^A/HDFG/IGF2BP3 axis	(140)
					METTL3/m^6^A/SEC62/IGF2BP1 axis	(141)
					METTL3/m^6^A/MYC axis	(143)
					METTL3/m^6^A/ARHGAP5 axis	(144)
	WTAP and RBM15	Writer	Upregulated	Poor prognosis; immune response	Unknown	(153–155)
	METTL14	Writer	Downregulated	Poor prognosis	METTL14/m^6^A/Wnt and PI3K-AKT axis	(156)
	FTO	Eraser	Upregulated	Poor prognosis	Unknown	(153, 155) (160),
	ALKBH3	Eraser	Upregulated	Poor prognosis	ALKBH3/m^1^A/ErbB2 and AKT1S1 axis	(161)
	ALKBH5	Eraser	Upregulated	Poor prognosis	ALKBH5/m^6^A/lncRNA NEAT1/EZH2 axis	(155, 162)
CRC	METTL3	Writer	Upregulated	Poor prognosis	METTL3/m^6^A/SOX2/IGF2BP2/tumor stemness axis	(145)
					METTL3/m^6^A/HK2 and GLUT1/IGF2BP2/3 axis	(148)
					METTL3/m^6^A/miR-1246/SPRED2/MAPK axis	(147)
					METTL3/m^6^A/p38-ERK or CCNE1 axis	(149, 150)
	METTL14	Writer	Downregulated	Poor prognosis	METTL14/m^6^A/lncRNA XIST/YTHDF2 axis	(158)
					METTL14/m^6^A/miR-375/YAP1 and SP1 axis	(159)
	FTO	Eraser	Downregulated	Poor prognosis	FTO/m^6^Am/tumor stemness axis	(146, 164)
	ALKBH5	Eraser	Downregulated	Poor prognosis	ALKBH5/m^6^A/lncRNA RP11/hnRNPA2B1/E-ligases/Zeb1 axis	(163)
	IGF2BP2	Reader	Upregulated	Poor prognosis	lncRNA LINRIS/IGF2BP2/MYC/glycolysis axis	(165)
	YTHDF3	Reader	Upregulated	Poor prognosis	lncRNA GAS5/YAP/YTHDF3 axis	(166)
	YTHDF1	Reader	Upregulated	Poor prognosis	YTHDF1/Wnt/β-Catenin and tumor stemness axis	(167)

GC, gastric cancer; CRC, colorectal cancer; writers, methyltransferases; erasers, demethylases; readers, the proteins that bind to methylation modifications; m^6^A, N^6^-methyladenosine; m^1^A, N^1^-methyladenosine; METTL3/14, methyltransferase-like3/14; WTAP, Wilms tumor 1-associated protein; RBM15, RNA-binding motif protein 15; FTO, fat mass and obesity-associated protein; ALKBH3/5, AlkB homolog 3/5; hnRNPA2/B1, heterogeneous nuclear ribonucleoprotein A2/B1; lncRNA, long non-coding RNA; miRNA, micro RNA; ZMYM1, zinc finger MYM-type containing 1; EMT, epithelial-mesenchymal transition; HDGF, hepatoma derived growth factor; SOX2, SRY (sex determining region Y)-box 2; HK2, Hexokinase 2; GLUT1, glucose transporter type 1; CCNE1, cyclin E1; XIST, X-inactive specific transcript; YAP1, Yes-associated protein 1; NEAT1, nuclear paraspeckle assembly transcript 1; EZH2,a subunit of the polycomb repressive complex; LINRIS, Long Intergenic Noncoding RNA for IGF2BP2 Stability; *has been identified at present.

However, there are some controversies and confusions in the RNA methylation: **i)** Binding mode of YTHDF proteins on different mRNAs or a single mRNA. **ii)** Affinity towards the m^6^A and m^6^Am sites by FTO. **iii)** mRNA stability affected by the m^6^Am modification. **iv)** The role of the m^1^A modification in RNA translation. **v)** Specificity of the targets regulated by the methylation regulators. **vi)** The distinguish expression signatures of both the writers and the erasers in certain type of GICs, and their downstream targets. **viii)** Inhibitors for METTL3 and readers.

## Author Contributions

GW and WH proposed these ideas and valuable comments, and QL drafted the manuscript. All authors contributed to the article and approved the submitted version.

## Funding

This work was supported in part by grants from the National Natural Science Foundation of China (82073869, 81701834, 81871994); Guangdong Basic and Applied Basic Research Foundation (2019A050510019, 2019B151502063); Guangdong Provincial Key Laboratory of Construction Foundation (2017B030314030, 2020B1212060034); Guangzhou Science and Technology Planning Program (202002020051, 201902020018); National Engineering Research Centre for New Drug and Drug ability Evaluation, Seed Program of Guangdong Province (2017B090903004).

## Conflict of Interest

The authors declare that the research was conducted in the absence of any commercial or financial relationships that could be construed as a potential conflict of interest.
